# Comparison of Knowledge and Attitudes Regarding Hepatitis B Among Healthcare Professionals in Pakistan

**DOI:** 10.7759/cureus.1049

**Published:** 2017-02-23

**Authors:** Asad Ali, Sara Khan, Saad M Malik, Muhammad Haris Iqbal, Muhammad Aadil

**Affiliations:** 1 Medicine, CMH Lahore Medical College and Institute of Dentistry; 2 Dentistry, CMH Lahore Medical College and Institute of Dentistry; 3 Community Medicine, Combined Military Hospital, Lahore, Pakistan; 4 Surgery, De' Montmorency College of Dentistry; 5 Department of Psychiatry and Mental Health, Rush University Medical Center

**Keywords:** doctors, dentists, nurses, medical students, dental students, hepatitis b virus, vaccination, attitude, awareness, healthcare professionals

## Abstract

**Aim:**

Hepatitis B virus (HBV) is a blood-borne infectious disease. It is one of the most common causes of end-stage liver disease, including cirrhosis and hepatocellular carcinoma. Healthcare professionals, including medical and dental students, are at a high risk of acquiring this infection. The aim of this study was to compare and contrast the knowledge and attitudes toward HBV infection amongst doctors, dentists, nurses, and undergraduate final year medical and dental students.

**Subjects and method:**

A cross-sectional study was carried out on a sample size of 381 medical professionals, which included doctors (59), dentists (77), nurses (71), final year medical students (126), and final year dental students (48) at Combined Military Hospital Lahore Medical College and Institute of Dentistry (CMH LMC). A questionnaire comprising 27 multiple choice questions was distributed amongst the groups mentioned above. The questionnaire aimed to assess basic knowledge, attitudes towards those infected, and knowledge about vaccination against HBV.

**Results:**

The total response rate was 88.8% (382/430 respondents returned the questionnaire). The mean ± standard deviation (SD) score for all healthcare professionals in knowledge was 15.54 ± 3.69 and attitude were 4.67 ± 1.37, which indicated that majority of the healthcare professionals were well informed about hepatitis B and generally exhibited positive attitudes. However, results revealed that medical students lacked adequate knowledge about various aspects of HBV infection, including modes of transmission and prevention methods against the disease. On the other hand, dental students were better informed and exhibited a more positive attitude towards the disease.

**Conclusion:**

According to the results of our study, medical students showed poor knowledge about hepatitis B disease, including its modes of transmission and the option of vaccination. Lack of knowledge contributed significantly to their negative attitudes towards those suffering from the disease, which has the potential to considerably affect the quality of patient care and the doctor-patient relationship. Major steps should be taken towards improving the curriculum followed at medical colleges in Pakistan. More emphasis should be laid on providing knowledge during early academic years and increasing the amount of clinical exposure. Frequent workshops and seminars should be organized in order to provide up-to-date knowledge about HBV infection and means of prevention to both healthcare professionals and students.

## Introduction

Hepatitis B virus (HBV) infection is a serious global health problem, with two billion people infected worldwide and 350 million suffering from chronic HBV infection [1]. It is counted amongst the most prevalent diseases in Pakistan [2]. According to the World Health Organization (WHO), about four million people in this country have already been exposed to HBV [2].

This infection is caused by a blood-borne pathogen called hepatitis B virus. It is one of the most common causes of end-stage liver diseases, including cirrhosis and hepatocellular carcinoma [3].

HBV is transmitted from one individual to another via direct blood to blood contact, from mother to child, and unprotected sexual intercourse. Risk factors associated with this infection include drug abuse, piercings, blood transfusions, dialysis, and dental procedures [2]. The virus is capable of surviving outside the body for prolonged periods of time [4]. According to WHO, the most common causes of HBV infection in Pakistan are the transfusion of unscreened blood, improper sterilization of medical instruments, and reuse of needles by healthcare workers [2].

HBV infection can be easily prevented. A vaccine, which is both safe and effective, is readily available [1]. The Expanded Programme on Immunization (EPI) was started by WHO in Pakistan in 1978. Hepatitis B vaccination was added to this program in 2002. However, routine vaccination coverage in Pakistan has not yet reached the optimal level, and a major portion of the population remains unvaccinated [5].

Healthcare professionals are at an increased risk of acquiring blood-borne infectious diseases [6-8]. These individuals are prone to needle-stick injuries during procedures, leading to inadvertent inoculation of infected blood [2]. Therefore, it is very important for these groups to follow a standard cross-infection protocol and be well aware of methods of prevention against HBV infection, including vaccination.

Moreover, due to the lack of hands-on clinical training and exposure in medical curricula in Pakistan, healthcare professionals, in general, and medical students, in particular, may fall prey to misconceptions leading to discriminatory behavior towards those infected [9]. This will lead to a negative attitude towards the disease and failure to provide optimal medical care to those patients suffering from infectious diseases, including HBV.

This survey was conducted to compare five groups of healthcare professionals including doctors, dentists, nurses, and undergraduate final year medical and dental students on the basis of their knowledge and attitude towards HBV infection.

## Materials and methods

### Study design

This cross-sectional survey was designed and conducted at Combined Military Hospital Lahore Medical College and Institute of Dentistry (CMH LMC) in Lahore, Pakistan, from February 2016 to March 2016. Permission to conduct this study was obtained from the CMH LMC Research Ethics Committee.

Stratified sampling technique was used to randomly select respondents. Healthcare providers were divided into groups based on their designation. A computer software was then used to generate random numbers. Data was collected using a total of 430 well-structured questionnaires which were distributed amongst potential respondents (doctors, dentists, nurses, and final year medical and dental students at the hospital and college, respectively). A sample size of 381 was obtained, which included doctors (59), dentists (77), nurses (71), final year medical students (126), and final year dental students (48). Informed consent was obtained from every individual included in this study after explaining the objectives of this research and assuring respondents of confidentiality.

Questionnaire

The questionnaire consisted of 27 questions aiming to assess awareness and attitudes towards HBV infection amongst the groups mentioned above. These questions were divided into three different categories. The first category of questions aimed to assess basic knowledge regarding HBV infection. Questions regarding the causative agent, modes of spreading, and risk factors associated with HBV infection were included. The second category dealt with assessing attitudes towards the disease as well as those infected with HBV infection. The last category was composed of questions dealing with prevention and vaccination against the disease. Permission to use this questionnaire for our study was granted by Amr Idris, author of the article “Hepatitis B Awareness Among Medical Students and Their Vaccination Status at Syrian Private University” [1], and was modified to our requirements.

### Data analysis

Data were analyzed using the SPSS version 21.0 (IBM SPSS Inc., Chicago, IL, USA). Mean (SD) was calculated for quantitative values and frequency (%) was calculated for demographics and response percentage on the questionnaire. Chi‑square test, one-way analysis of variance (ANOVA), and Tukey's honest significant difference (HSD) test were applied for analysis and comparison of categorical data.

## Results

The questionnaire was completed by 381 of the 430 healthcare professionals (response rate: 88.8%). The mean age of the respondents was 24.17 ± 5.20 years, and 65.6% were female, and 34.4% were male. The demographic data and vaccination status of the sample are presented in Table 1. 

**Table 1:** Demographics and vaccination status of respondents

Variable		N (%) (N=381)
Age category (years)	<30	346 (90.8)
	30-40	26 (6.8)
	>40	9 (2.4)
Gender		
	Male	131 (34.4)
	Female	250 (65.6)
Healthcare group		
	Medical doctor	59 (15.5)
	Dentist	77 (20.2)
	Nurse	71 (18.6)
	Medical student	126 (33.1)
	Dental student	48 (12.6)
Vaccinated against hepatitis B	Yes	287 (75.3)
	No	94 (24.7)
Tested for hepatitis B	Yes	306 (80.3)
	No	75 (19.7)

The level of knowledge about HBV infection was satisfactory with a mean ± SD score of 15.5 ± 3.69 out of a total possible score of 22. The level of knowledge was higher in doctors and dentists (18.22 ± 2.69 and 18.17 ± 1.76, respectively) and was lowest in medical students (11.57 ± 2.16). The attitude score followed the same pattern, being highest in doctors (5.37 ± 1.22) and lowest in medical students (3.79 ± 1.21) out of a total score of seven. Females scored higher on knowledge and attitude than males and participants in older age groups had better knowledge and attitude levels than the younger respondents. Table 2 shows the comparison of healthcare groups, age, and gender in relation to their knowledge and attitude scores by one-way ANOVA.

**Table 2:** Comparison of healthcare groups by one way ANOVA

*Designation*	*Knowledge*		*Attitude*	
	Mean SD		Mean SD	
*Doctor (n=59) *	18.22	2.69	5.37	1.22
*Dentist (n=77) *	18.17	1.76	5.20	1.20
*Nurse (n=71)*	17.17	1.96	5.25	1.08
*Medical student (n=126)*	11.57	2.16	3.79	1.21
*Dental student (n=48)*	16.00	3.35	4.38	1.27
*p value*	0.000		0.000	
*Gender*				
*Male*	13.82	3.65	4.32	1.30
*Female*	16.43	3.39	4.85	1.37
*p value*	0.000		0.000	
*Age group (years)*				
*< 30*	15.27	3.73	4.60	1.36
*30-40*	17.92	1.85	5.31	1.41
*> 40*	18.67	1.58	5.56	1.01
*p value*	0.000		0.005	
*Overall*	15.54	3.69	4.67	1.37

**Table 3:** Correct answers for some knowledge questions for different healthcare groups

Question	Doctor N (%)	Dentist N (%)	Nurse N (%)	Medical Student N (%)	Dental Student N (%)	Total N (%)	P value
Have you heard of hepatitis B?	58 (98.3)	74 (96.1)	70 (98.6)	111 (88.1)	47 (97.9)	360 (94.5)	0.004
Causative agent of hepatitis B?	58 (98.3)	77 (100)	71 (100)	73 (57.9)	47 (97.9)	326 (85.6)	0.000
Mode of spread of hepatitis B?	51 (85.6)	55 (70.8)	58 (81.0)	44 (34.9)	32 (66.7)	237 (62.2)	0.000
Risk factors that may be the cause of hepatitis B?	48 (81.4)	63 (82.2)	52 (72.8)	22 (17.2)	25 (52.1)	210 (55.0)	0.000
Signs and symptoms of Hepatitis B?	55 (93.2)	65 (84.4)	70 (98.0)	17 (13.5)	28 (58.3)	235 (61.7)	0.000
What does chronic Hepatitis B lead to?	15 (25.4)	15 (19.5)	22 (31.0)	2 (1.6)	6 (12.5)	60 (15.7)	0.000
Is it preventable?	56 (94.9)	76 (98.7)	70 (98.6)	51 (40.5)	34 (70.8)	287 (75.3)	0.000
Vaccine available?	57 (96.6)	73 (94.8)	70 (98.6)	49 (38.9)	35 (72.9)	284 (74.5)	0.000
Does vaccine provide protection?	57 (96.6)	73 (94.8)	70 (98.6)	49 (38.9)	35 (72.9)	303 (79.5)	0.000
Hepatitis B vaccine can be safely administered with other vaccines	50 (84.7)	70 (90.9)	60(84.5)	115 (91.3)	44 (91.7)	339 (89.0)	0.427

Regarding attitude scores, 18.9% of the participants believed that chronic infection with hepatitis B is shameful. Only 48.3% did not mind sharing food with the hepatitis B infected individual, and 39.9% of the population had concerns about shaking hands or hugging a patient infected with Hepatitis B virus. A summary of attitude scores for different healthcare groups is presented in Table 4.

**Table 4:** Correct answers for some attitude questions for different healthcare groups

Question	Doctor N (%)	Dentist N (%)	Nurse N (%)	Medical Student N (%)	Dental Student N (%)	Total N (%)	P value
Would you accept Hepatitis B patient in the same class as yours?	51 (86.4)	68 (88.3)	62 (87.3)	82 (65.1)	31 (64.6)	294 (77.2)	0.000
You don’t mind sharing food with Hepatitis B patient	40 (67.8)	36 (46.8)	30 (42.3)	62 (49.2)	16 (33.3)	184 (48.3)	0.005
Chronic infection with hepatitis B is shameful	53 (89.8)	68 (88.3)	70 (98.6)	83 (65.9)	35 (72.9)	309 (81.1)	0.000
Caring for hepatitis B patient makes you uncomfortable	50 (84.7)	68 (88.3)	61 (85.9)	71 (56.3)	38 (79.2)	288 (75.6)	0.000
Shaking hands/hugging hepatitis B patient makes you uncomfortable	37 (62.7)	52 (68.8)	59 (83.1)	50 (39.7)	30 (62.5)	229 (60.1)	0.007
Hepatitis infected doctors/dentists/nurses should be allowed to work	23 (39.0)	41 (53.2)	49 (69.0)	60 (47.6)	28 (58.3)	201 (52.8)	0.240
Dental/medical personnel should refuse treatment to hepatitis B infected person	50 (84.7)	72 (93.5)	69 (97.2)	63 (50.0)	40 (83.3)	294 (77.2)	0.000

Comparison of healthcare groups with respect to their knowledge and attitude scores showed that the difference in knowledge and attitude scores between doctors and medical students was statistically significant (p < 0.05). The difference was also statistically significant between dentists and dental students. The difference in knowledge and attitude scores between doctors and dentists and doctors and nurses was not statistically significant (Table 5).

**Table 5:** Comparison of healthcare groups by Tukey’s HSD test

	*Knowledge*	*Attitude*
*Doctor vs Dentist*	1.000	0.930
*Doctor vs Medical Student*	0.000*	0.000*
*Dentist vs Dental Student*	0.000*	0.002*
*Doctor vs Nurse*	0.079	0.980

Participants whose knowledge was weaker were more likely to get a low attitude score, and those who scored high on knowledge were more likely to show a high attitude score (p = 0.01). A significant association between the knowledge score and attitude score was shown by regression analysis (r = 0.4, p = 0.01). The majority of the participants were vaccinated (75.3%) against HBV and 82.7% believed that healthcare professionals should receive hepatitis B vaccination. Lack of motivation was the major reason for not getting vaccinated (64.0%). Most (82.4%) voted in favor of providing training programs regarding the occupational risk of HBV to both medical and dental students.

## Discussion

According to our study, healthcare professionals are well aware of the existence of HBV infection and acknowledge the occupational risk associated with this disease. However, it was found that final year medical students have poor knowledge regarding several aspects of HBV infection including modes of transmission, risk factors, and the availability of an effective vaccine. In contrast to these findings, it was found that final year dental students are better informed about the various modes of spread of infection and believe that vaccination against HBV is available and provides protection.

Blood transfusions are considered to be one of the most important risk factors associated with HBV transmission [2]. However, in our study, a total of 55.6% medical students think otherwise. On the other hand, 97.9% of dental students were well aware of this particular mode of transmission of HBV infection. A possible reason for the lack of awareness among medical students is the absence of an integrated system of education. Integration allows medical students to engage in academic activities which can be connected to their practical lives. This is well supported by a study conducted in Karachi, Pakistan, which concluded that students had a better command of integrated subjects as opposed to courses taught without integration [9]. In contrast, dental students are involved in direct patient care from their early years in dental school. Hence, by the time they reach their final year, they have sufficient clinical exposure and are much more confident and are capable of handling patients presenting with infectious diseases and following standard cross-infection protocols. This is reflected in the results of our study. Older healthcare professionals showed better attitudes and knowledge scores. A study conducted at Al-Farabi College of Dentistry and Nursing, Riyadh, Saudi Arabia stated that dental students from a higher academic year exhibited a more positive attitude towards infection control practices as compared to those from lower academic years [10]. This may be due to both theoretical and practical learning over the years which eventually lead to an improvement in the attitudes of dental students towards patients suffering from infectious diseases. Hence, it is important to provide a similar standardized clinical environment to medical students from their first year all the way to final year. Reinforcement of knowledge of the vast disease spectrum over the years will lead to adequate awareness amongst medical students once they reach the final year of medical school. 

As far as attitude towards those infected with HBV infection is concerned, a significant number of medical students do not feel comfortable making physical contact with those infected (60.3%). This may be due to their lack of awareness regarding the various modes of transmission of HBV. If this misconception is not guarded against, it may lead to refusal of treatment and a decline in the quality of medical care provided [8]. In contrast, dental students (88.3%) showed more willingness towards treating patients with HBV. This is consistent with the findings of Nuttall and Gilbert: 84.3% of final-year dental students in the United Kingdom were willing to treat patients with HBV [11].

Therefore, regular workshops and seminars should be held to provide up-to-date knowledge to both medical and dental students, remove misconceptions and instill positive doctor-patient attitudes regarding the disease and its mode of spread.

Our study reveals that the majority of our targeted population was aware of the occupational risk of being infected with HBV infection (77.0%). However, in this survey, medical students again lacked overall knowledge of the occupational risk of acquiring HBV infection. Needle stick injury is one of the most common causes of HBV infection amongst doctors and dentists. According to a study, 8,617 HBV infections occur every year in Egypt as a result of needlestick injuries [12]. Another study conducted at Kathmandu Medical College and Teaching Hospital concluded that the knowledge of healthcare professionals regarding the risks associated with needlestick injuries was inadequate [13].

Our study shows that more than 50% of the students (medical 66.7% and dental 77.1%) had received a Hepatitis B vaccination, but a large number of them (medical 40% and dental 72.9%) did not have enough knowledge about the vaccine itself. These findings are consistent with a survey conducted in Pakistan, which found that knowledge regarding hepatitis B vaccine was not satisfactory amongst the study groups [9]. Results show that doctors, dentists, and nurses firmly believe that healthcare professionals should receive vaccination against HBV; however, not everyone was vaccinated against HBV and were not being regularly tested for HBV. Considering healthcare professionals work in proximity to those suffering from infectious diseases, it is imperative for each and every individual to receive vaccination as well as undergo regular testing and monitor antibody titer [14]. Regular follow-up testing is also very important for early detection and subsequent timely treatment of those inadvertently infected by HBV. Every hospital should be responsible for providing vaccinations against HBV to all its healthcare professionals as well as for ensuring regular testing [15]. Moreover, both medical and dental students should be encouraged to get vaccinated against HBV before they get involved in direct patient care [16]. Further studies should be conducted on occupational risks associated with HBV infection and subsequent post-exposure prophylaxis.

## Conclusions

According to the results of our study, medical students showed poor knowledge about hepatitis B disease, including its modes of transmission and the option of vaccination. Lack of knowledge contributed significantly to their negative attitudes towards those suffering from the disease, which has the potential to considerably affect the quality of patient care and the doctor-patient relationship. Major steps should be taken towards improving the curricula followed at medical colleges in Pakistan. More emphasis should be laid on providing knowledge during early academic years and increasing the amount of clinical exposure. Frequent workshops and seminars should be organized to provide up to date knowledge about HBV infection and means of prevention to both healthcare professionals and students.
